# The proper timing of virtual reality experience for reducing preoperative anxiety of pediatric patients: A randomized clinical trial

**DOI:** 10.3389/fped.2022.899152

**Published:** 2022-09-13

**Authors:** Jung-Hee Ryu, Dayoung Ko, Ji-Won Han, Jin-Woo Park, Aesun Shin, Sung-Hee Han, Hyun-Young Kim

**Affiliations:** ^1^Medical Virtual Reality Research Group, Department of Anesthesiology and Pain Medicine, Seoul National University College of Medicine, Seoul, South Korea; ^2^Department of Anesthesiology and Pain Medicine, Seoul National University Bundang Hospital, Seongnam, South Korea; ^3^Department of Pediatric Surgery, Seoul National University Children’s Hospital, Seoul, South Korea; ^4^Department of Preventive Medicine, Seoul National University College of Medicine, Seoul, South Korea; ^5^Department of Surgery, Seoul National University College of Medicine, Seoul, South Korea

**Keywords:** anxiety, distress, pediatric patients, virtual reality, anesthesia

## Abstract

**Background:**

The virtual reality (VR) experience of an operation room (OR) prior to anesthesia and surgery has been known to reduce the anxiety and distress of pediatric patients. However, the proper timing needed for this is unknown. This randomized clinical study aimed to evaluate the proper timing of a VR tour of an OR (a few days before vs. immediately before anesthesia) to reduce the anxiety in a pediatric patient undergoing elective surgery.

**Methods:**

The children from the ages of 4–10 years old were randomly divided into three groups. The control group received standard verbal information about the process of anesthesia and surgery 10 min before anesthesia. The VR A group experienced a VR tour at the outpatient clinic a few days before anesthesia, whereas the VR B group experienced the tour 10 min before anesthesia at the reception area of the OR. The 4-min VR video used in this study showed the experience of Pororo, an animation character, entering the OR and undergoing anesthesia. We evaluated the anxiety of children using the modified Yale preoperative anxiety scale (m-YPAS), the anxiety of caregivers using Beck anxiety inventory (BAI), and caregivers’ satisfaction.

**Results:**

The m-YPAS of the VR B group was significantly lower than that of the control and VR A groups (*p* = 0.001), whereas there was no statistically significant difference in BAI (*p* = 0.605) among the 3 groups. The score of caregivers’ satisfaction with the overall process of anesthesia and surgery was higher in VR A group than in the control and VR B groups (*p* = 0.054).

**Conclusion:**

The VR experience of an OR immediately before anesthesia was more effective than standard verbal information or a VR tour at the outpatient clinic a few days before anesthesia in reducing the anxiety and distress of children prior to surgery.

**Clinical trial registration:**

[https://cris.nih.go.kr/cris/search/detailSearch.do/20773], identifier [KCT0006845].

## Introduction

Surgery and anesthesia are extremely stressful events for pediatric patients and their parents. The incidence of preoperative anxiety has been reported to be nearly 60% for pediatric patients, which may be due to fear of pain, unfamiliarity, and even death (1). Preoperative anxiety usually presents itself as numerous forms of behaviors, including agitation, crying, shivering, fighting, and attempting to escape from health providers (1); these may delay the process of anesthesia induction. Furthermore, children with high preoperative anxiety have been found to show higher postoperative pain and emergence delirium (2). Several attempts have been made to reduce children’s preoperative anxiety, including premedication, parental presence, information, and distraction (3, 4).

In the modern era of digital technology, virtual reality (VR) was originally used for entertainment purposes, but it has recently been introduced into many medical fields. One of the clinical applications of VR technology is to educate pediatric patients and address their preoperative anxiety before anesthesia and surgery (5). This is because preoperative anxiety has a strong psychological component, which may allow VR to direct children’s attention to the simulated environment (5). VR enables pediatric patients to immerse themselves in this virtual environment through visual, auditory, tactile, and olfactory sensations, thus focusing their attention away from the unknown and fearful hospital situation (5). Additionally, preoperative anxiety of pediatric patients may arise from the unfamiliarity or strangeness about the unknown event or space and they can learn from VR experience of hospital environment.

However, there is little information on the proper timing of the VR experience to reduce preoperative anxiety in pediatric patients. In this study, pediatric patients undergoing elective surgery experienced VR of the operation room (OR) at the outpatient clinic a few days before anesthesia or 10 min before anesthesia at the reception area of the OR. Preoperative anxiety, compliance during induction of anesthesia, and anxiety of the caregiver(s) were evaluated to establish the optimal timing for undergoing the VR experience.

## Methods

### Study

This study was a prospective randomized clinical trial conducted in Seoul National University Children’s Hospital between September 2017 and February 2019. This trial was approved by the institutional review board of Seoul National University Hospital (IRB 1706-168-863), and the protocol was registered (Protocol no. KCT0006845). We obtained the informed consent, with detailed instructions, from the parents of all participants, and children aged 7 years or older signed additional agreements.

### Patients

The patients between the ages of 4 and 10 years who underwent operations in pediatric surgery—including benign soft mass excision, inguinal hernia repair, central catheter insertion, and frenotomy—were enrolled in this study. All of them presented a physical status of class I or II from the American Society of Anesthesiology physical status classification. Children with premature birth history, chronic disease, hearing disorders, epilepsy, and previous experience with anesthesia were excluded.

### Randomization and intervention

We randomized the patients into three groups using a computer-generated randomization code (Random Allocation Software Version 1.0): the control, VR A (VR tour at the outpatient clinic a few days before anesthesia), and VR B (VR tour 10 min before anesthesia at the reception area of the OR) groups.

Allocation was performed at the time of decision for operation by the independent researcher. Children in the control group received standard information concerning the process of anesthesia and operation 10 min before anesthesia. Children in the VR A group experienced a VR tour on the process of anesthesia at the outpatient clinic when the operation was decided, whereas children in the VR B group received this tour in the reception area of the operating theater 10 min before anesthesia. The intervention was performed by surgeons of the research team.

### Virtual reality experience

**FIGURE 1:**
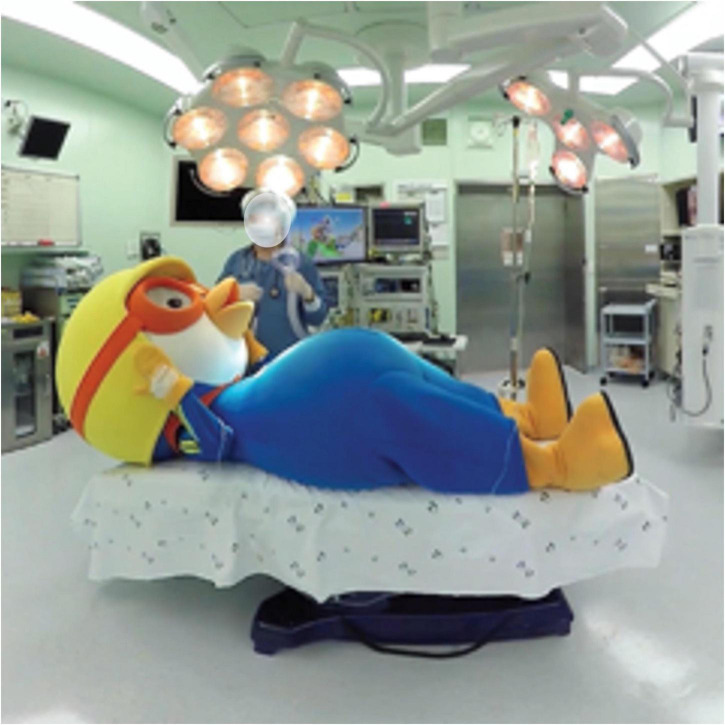
Virtual reality tour. Pororo is transported to the operating room area. Monitoring devices, including ECG leads, non-invasive blood pressure cuff, and pulse oximeter, are attached.

The VR tour was offered as a 4-min video using a head-mounted display, an Oculus Go (Oculus VR) produced in collaboration with IONIX (Seongnam, South Korea) and a VR producing company (The VR, Seoul, South Korea). The animation characters—Pororo and other characters from the film “Pororo The Little Penguin” (©ICONIX/OCON/EBS/SKBroadband)—were described as patients who were scheduled for surgery and toured the operating theater (Figure 1). The patients could experience the whole process through VR, from intravenous catheterization to entering the theater. During the tour, Pororo explained all the preoperative processes in detail but in a friendly manner, with the goal of reducing the children’s anxiety.

### Induction of anesthesia

Induction of anesthesia was conducted by anesthesiologists with at least 2 years of experience. Thiopental sodium 5 mg/kg was intravenously administered, followed by mask ventilation with sevoflurane in oxygen (5 L/min) applied after the disappearance of the eyelid reflex. Standard monitoring—including electrocardiogram (ECG), non-invasive blood pressure cuff, and pulse oximeter—was placed. Rocuronium 0.6 mg/kg was administered, and endotracheal intubation was undertaken. Maintenance of anesthesia was carried out with sevoflurane with medical air in oxygen (FiO_2_ 0.5).

### Outcome measurement

The modified Yale Preoperative Anxiety Scale (m-YPAS) score for children was measured by a blinded observer in the reception area at the operating theater immediately before entering; the children and caregivers were not blinded. The m-YPAS scales comprise five domains: activity, vocalizations, emotional expressivity, state of arousal, and use of parents (6). The total score of the m-YPAS is 100, where high scores indicate high levels of patients’ anxiety. We investigated the caregiver’s anxiety using the Beck Anxiety Inventory (BAI) after the child entered the OR (7). BAI consists of a self-reporting list of 21 items with a 4-point anxiety scale (0; none, 3; severe) (7). Total scores are calculated by summing scores for each item, ranging from 0–63. We also investigated the caregivers’ satisfaction with the overall process of anesthesia and surgery using a numeric rating scale (0, very dissatisfied; 50, very satisfied) a week after the operation performed.

### Statistical analysis

SPSS version 25.0 was used for statistical analysis. All continuous variables are presented as median values within the interquartile range. A normality test was first performed to determine the significance of continuous variables. Fisher’s exact test was used to analyze continuous variables, and either the Mann–Whitney *U* test or the Kruskal–Wallis test were used to analyze categorical variables. *P* < 0.05 were considered statistically significant. After the Kruskal–Wallis test, we performed a Mann–Whitney *U* test with Bonferroni correction. A full analysis set was used for data analysis. *P* < 0.025 were considered to be statistically significant.

## Results

**FIGURE 2:**
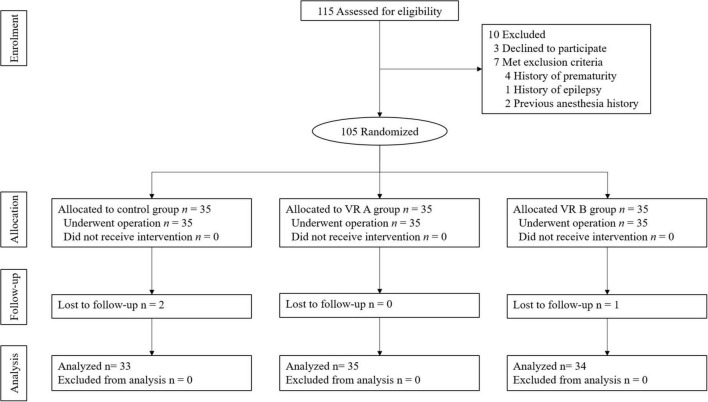
CONSORT diagram for the trial.

Out of 115 children, after excluding those who met the exclusion criteria (Figure 2), a total of 105 patients were randomized, and assigned to the control, VR A, or VR B groups. Therefore, each group included 35 patients. During the study period, caregivers of three pediatric patients (two in the control group and one in the VR B group) were lost to follow-up after the surgery without satisfaction scores and excluded from the final analysis (Figure 2).

Patients’ characteristics are presented in Table 1. The median age of the patients was 5.0 (IQR 5.0; 6.0) years, 6.0 (IQR 4.0; 7.5) years, and 5.0 (IQR 5.0; 6.5) years in the control, VR A, and VR B groups, respectively.

**TABLE 1:** Patient characteristics.

	Control group (*n* = 35)	VR A group (*n* = 35)	VR B group (*n* = 35)
Age (year)	5.0 [5.0;6.0]	6.0 [4.0;7.5]	5.0 [5.0;6.5]
Sex, M/F, *n* (%)	15/50 (43/57)	17/18 (49/51)	16/19 (46/54)
Reason for the operation, *n* (%)			
Hernia	24 (68.6)	20 (57.1)	15 (42.9)
Benign mass	10 (28.6)	10 (28.6)	12 (34.3)
Central catheter insertion	1 (2.9)	1 (2.9)	4 (11.4)
Tongue tie	0 (0.0)	4 (11.4)	4 (11.4)
Anesthesia time, min	55.5 [45.0; 72.5]	60.0 [42.5; 75.0]	50.0 [40.0; 62.5]
Operation time, min	35.0 [20.0; 45.0]	32.0 [17.5; 42.5]	20.0 [10.0; 37.5]
Hospital stay, day	1.0 [1.0; 3.0]	1.5 [1.0; 2.0]	1.0 [1.0; 2.0]

Control group, Standard verbal information concerning the process of anesthesia 10 min before anesthesia; VR A group, a virtual reality tour on the process of anesthesia at the outpatient clinic when the operation was decided; VR B group, a virtual reality tour on the process of anesthesia tour in the reception area of operating theater 10 min before anesthesia. Values are presented as median [IQR] or n (%).

Regarding the preoperative anxiety of children, there was a significant difference in m-YPAS among the three groups (Table 2, *p* = 0.001). The m-YPAS score was significantly lower in the VR B group than in the control group (control vs. VR B, *p* = 0.001). However, there was no significant difference in m-YPAS score between the control and VR A groups (control vs. VR A, *p* = 0.413) and between the VR A and VR B groups (VR A vs. VR B, *p* = 0.070).

**TABLE 2:** Preoperative anxiety, compliance during induction of anesthesia, caregivers’ distress, and satisfaction.

		Control group (*n* = 33)	VR A group (*n* = 35)	VR B group (*n* = 34)	*P*-value
Patients	m-YPAS	50.0 [38.3;60.0]	38.3 [25.8;54.2]	22.5 [13.3;45.0]	0.001*
Caregiver	BAI	5.0 [2.0; 8.0]	5.0 [1.0; 9.0]	6.0 [3.0; 8.5]	0.605
	Satisfaction	34.0 [28.0;41.0]	39.0 [36.0;46.0]	37.0 [32.0;45.0]	0.054

Control group, Standard verbal information concerning the process of anesthesia 10 min before anesthesia; VR A group, a virtual reality tour on the process of anesthesia at the outpatient clinic when the operation was decided; VR B group, a virtual reality tour on the process of anesthesia tour in the reception area of operating theater 10 min before anesthesia. m-YPAS, modified Yale Preoperative Anxiety Scale; Beck Anxiety Inventory. Values are presented as median (IQR). *Control vs. VR B, p = 0.001, control vs. VR A, p = 0.413, VR A vs. VR B, p = 0.070.

Regarding the anxiety of caregivers, there was no significant difference in BAI (*p* = 0.605) among the three groups. The result of BAI was 5 or 6, which can be interpreted as normal or no anxiety. Median satisfaction scores of caregivers on the overall process of anesthesia and surgery were 34, 39, and 37 in the control, VR A, and VR B groups, respectively, showing borderline significance (*p* = 0.054). Comparing the caregivers’ satisfaction between the control and VR A groups, the VR A group had better satisfaction than that of the control group (control vs. VR A, *p* = 0.014).

## Discussion

This is the first clinical trial investigating the proper timing of VR experience for reducing children’s preoperative anxiety. A VR tour of the OR immediately before anesthesia was more effective in relieving preoperative anxiety than a VR tour a few days before surgery at the outpatient clinic. However, there were no significant differences in compliance during induction of anesthesia, anxiety, and satisfaction of caregivers among the groups.

The results of the m-YPAS scores can be explained by the fact that the preoperative anxiety of children is usually intense in the reception area of the OR before induction of anesthesia (8). The effectiveness and feasibility of a VR experience on the anxiety of pediatric patients have been investigated in several clinical trials (9–14). Additionally, Koo et al. (15) performed a systemic review and meta-analysis to evaluate the effect of VR on preoperative anxiety, which found this effect to be more significant in pediatric patients than in adult patients. Another systemic review and meta-analysis also showed that VR distraction was effective in reducing pain and anxiety in pediatric patients (5). Children in these studies experienced VR immediately before anesthesia instead of a few days before surgery at the outpatient clinic. The results of this study affirm that a VR tour of the OR was more effective at reducing preoperative anxiety than the standard verbal information, as well as concluding that the proper timing to have the VR experience to reduce preoperative anxiety in children was immediately before anesthesia.

Caregivers’ anxiety was scored since it was considered to be closely related to the preoperative anxiety of children (16). The previous study showed that parental co-experience of the VR tour with children via mirroring of the display immediately before anesthesia was effective in reducing preoperative anxiety in both children and parents (17). However, in our study, only children received interventions. It is interesting to note that caregivers’ satisfaction scores about the overall process of anesthesia and surgery were highest in those that experienced a VR tour a few days before surgery at the outpatient clinic—although this difference did not reach statistical significance. This phenomenon may be interpreted as caregivers are likely to be satisfied when their children experience the VR of the OR at the outpatient clinic in advance. In most cases, there was no place to experience the VR tour in the OR reception area. In contrast, children in the OPD clinic could experience VR more comfortably than in the reception area. This environmental factor might affect the parents’ satisfaction.

There are a few limitations to be considered. First, the anxiety levels of children were evaluated once, right before entering the OR, without assessing the baseline value. However, it has been demonstrated in former studies comparing the baseline value that the baseline level of anxiety of pediatric patients is comparable between the control and VR intervention groups (10, 11, 14, 17). Second, pediatric patients in the VR A group received intervention a few days before anesthesia and surgery. However, the exact time for the intervention was not strictly controlled, ranging from 2 to 4 days preoperatively, depending on the schedule of the outpatient clinic. For this reason, reflections on the contents of the VR experience may vary among the children. This study has only one group of patients that received standard information. However, to evaluate the proper timing of the non-pharmacological technique for reducing preoperative anxiety of pediatric patients, one more correspondent group of patients who may receive the standard information days before surgery and not a few minutes before surgery is needed (18).

In conclusion, this randomized and controlled trial with pediatric patients suggests that the VR experience of the OR before anesthesia and surgery is effective for reducing the preoperative anxiety of children and that the proper timing to have this experience is immediately before anesthesia and surgery.

## Data availability statement

The raw data supporting the conclusions of this article will be made available by the authors, without undue reservation.

## Ethics statement

The studies involving human participants were reviewed and approved by Institutional Review Board of Seoul National University Hospital. Written informed consent to participate in this study was provided by the participants or their legal guardian/next of kin.

## Author contributions

DK and J-WP: data collection. DK, J-WH, and AS: data analysis and interpretation. J-HR and DK: major contribution in writing the manuscript. S-HH, and H-YK: conception and design analysis. All authors have read and agreed to the published version of the manuscript.
